# Therapeutic outcomes of enzalutamide-docetaxel combination versus docetaxel monotherapy in post-sequential androgen receptor axis-targeted therapy failure metastatic castration-resistant prostate cancer

**DOI:** 10.3389/fonc.2025.1608786

**Published:** 2025-08-15

**Authors:** Zhiyu Zhang, Yanhang Yu, Chuanao Zhang, Jianglei Zhang, Xuefeng Zhang, Jun Ouyang

**Affiliations:** Department of Urology, The First Affiliated Hospital of Soochow University, Suzhou, China

**Keywords:** enzalutamide, docetaxel, metastatic castration-resistant prostate cancer (mCRPC), progression-free survival, adverse effects, prostate-specific antigen

## Abstract

**Objective:**

This study aimed to evaluate the therapeutic efficacy and adverse effects of combining enzalutamide with docetaxel versus using docetaxel alone in treating metastatic castration-resistant prostate cancer (mCRPC) that progresses after treatment with abiraterone followed by enzalutamide.

**Methods:**

A retrospective analysis involved 67 mCRPC patients at the First Affiliated Hospital of Soochow University’s Urology Department between October 2021 and August 2023. All experienced disease progression after treatment with abiraterone and enzalutamide. Patients were either in the study group, receiving enzalutamide and docetaxel, or in the control group, treated with docetaxel alone. Prostate-specific antigen (PSA) levels, imaging changes, and common adverse reactions were compared.

**Results:**

The study group showed a more significant reduction in PSA levels (≥50%) and improved outcomes in bone and lymph node metastases than the control group (P < 0.05). The median PSA progression-free survival (PFS) was longer for the study group at 193 days (95% CI: 174–207) versus 127 days (95% CI: 114–160) for the control group. Similarly, the median PFS for bone metastases was 271 days (95% CI: 265–274) in the study group, compared to 185 days (95% CI: 183–265) in the control group. For lymph node metastases, PFS was 265 days (95% CI: 194–274) versus 183 days (95% CI: 180–189), respectively, all statistically significant (P < 0.05). Visual analog scale scores decreased significantly post-treatment in both groups (P < 0.05), with more pronounced pain relief in the study group; median scores were 2 (IQR, 1–3) versus 3 (IQR, 3–5; P < 0.05). No Grade 3 or higher adverse reactions occurred, although the study group had more malaise, lumbago, and backache (P < 0.05). There were no significant differences in myelosuppression, gastrointestinal issues, liver dysfunction, neurological symptoms, edema, rash, or high blood pressure between groups (P > 0.05).

**Conclusion:**

Combining enzalutamide with docetaxel is more effective than docetaxel alone for treating mCRPC after abiraterone and enzalutamide, providing better PSA-PFS and improved metastasis outcomes, along with better pain relief. Though the combination resulted in more adverse effects, no severe reactions (Grade 3 or higher) were observed, indicating good tolerability and clinical potential.

## 1 Introduction

Prostate cancer (PCa) is the second most common malignant tumor worldwide and the fifth leading cause of cancer-related deaths among men (1). Notably, it frequently metastasizes, predominantly to the bones, with approximately 90% of patients with advanced PCa experiencing bone metastases (2). Untreated metastatic PCa typically responds well to androgen deprivation therapy (ADT), also known as metastatic hormone-sensitive prostate cancer (mHSPC) (3). However, most cases eventually progress to metastatic castration-resistant prostate cancer (mCRPC), complicating treatment significantly (4). To address this, novel hormonal therapies (NHTs) have been developed (5–7). Abiraterone was the first NHT used for mCRPC and is recommended as a first-line treatment in numerous studies and guidelines with widely recognized efficacy (8–10). However, abiraterone resistance is a major clinical issue. In addition to abiraterone, enzalutamide (MDV3100) is another first-line NHT for mCRPC (8–10). Some researchers advocate the use of enzalutamide in abiraterone-resistant mCRPC, whereas others opt for docetaxel as a subsequent treatment (11). When both abiraterone and enzalutamide fail, docetaxel chemotherapy is the preferred treatment option. With advances in pharmacology and clinical applications, combinations of various NHTs with docetaxel chemotherapy have been proposed (12, 13). Recent studies have suggested considering a combination of enzalutamide and docetaxel for patients with mCRPC who progress after responding to enzalutamide, although this combined approach is not yet widely used and requires further investigation (13).

Based on this background, we included 67 mCRPC patients who progressed after effective treatment with abiraterone followed by enzalutamide. We investigated the effectiveness of subsequent treatment using enzalutamide combined with docetaxel chemotherapy versus docetaxel alone. This study aims to provide clinical insights for treatment strategies.

## 2 Materials and methods

### 2.1 Study design

This was a retrospective cohort study.

### 2.2 Clinical data

We retrospectively collected the clinical data of 67 patients with prostate cancer treated between October 2021 and August 2023 at the Urology Department of the First Affiliated Hospital of Soochow University. The inclusion criteria were as follows: (1) patients who underwent radical prostatectomy with postoperative pathological confirmation of malignant prostate tumors; and (2) patients initially treated with abiraterone and prednisone at the mHSPC stage progressed to mCRPC and showed progression after effective enzalutamide treatment. “Effective treatment with enzalutamide” was defined as a regimen of ADT plus oral enzalutamide 160 mg once daily, which resulted in a decrease in prostate-specific antigen (PSA) levels and/or imaging improvement. The exclusion criteria were as follows : (1) patients who did not consistently follow the treatment regimen, changed medications, discontinued treatment, or independently used other anticancer drugs; (2) patients who did not comply with regular follow-ups or had incomplete follow-up data; and (3) concurrent tumors at other sites.

### 2.3 Therapeutic schedule

Qualified patients were briefed on the treatment options, and subsequent therapies were chosen in consultation with the patients and their families. In addition to ADT, the study group (Group DP+MDV3100) included 38 patients who received oral enzalutamide at 160 mg once daily (continued usage) combined with docetaxel at 75 mg/m² and oral prednisone at 5 mg twice daily. This regimen was administered in 21-day cycles for eight cycles. The control group (Group DP) consisted of 29 patients treated with docetaxel at 75 mg/m² and oral prednisone at 5 mg twice daily in the same 21-day cycle regimen for eight cycles. To prevent bone degradation, patients were treated with 4 mg of zoledronic acid monthly. Patients underwent monthly tests for serum PSA, liver and kidney function, and electrolyte levels. Chest and abdominal computed tomography (CT), pelvic magnetic resonance imaging, and emission computed tomography were performed every 3 months.

### 2.4 Observation metrics

Patient outcomes post-treatment were evaluated through several criteria: the percentage of cases where PSA levels declined by ≥50% (the lowest PSA value recorded during the study), pain scores assessed using the Visual Analog Scale (VAS) (the lowest VAS score during the study period), and improvements observed in imaging of bone and lymph node metastases. Progression-free survival (PFS) was assessed based on PSA levels and imaging studies. The follow-up period is scheduled to be completed in December 2024. Endpoint events included PSA antigen, bone metastasis, and lymph node metastasis. Throughout the observation period, regular checks were conducted for blood counts, liver and kidney functions, and electrolyte levels. Any adverse events occurring during medication were recorded and evaluated according to the CTCAE version 5.0 grading standards, with appropriate management applied (14).

### 2.5 Statistical analysis

Data analysis was conducted using R version 4.2.1, leveraging the “stats [4.2.1],” “survival [3.3.1],” “survminer [0.4.9],” and “ggplot2 [3.4.4]” packages. Categorical data are represented as frequencies (%), while continuous data are shown as the mean ± standard deviation. For numerical variables, if the data met the assumptions of a normal distribution and homogeneity of variance, a t-test was used for group comparisons. If the data were normally distributed but violated the homogeneity of variance assumption, Welch’s t-test was applied. Non-normally distributed data were analyzed using the Wilcoxon test. For categorical variables, when the expected frequency was greater than five, and the total sample size was 40 or more, the chi-square test was used for group comparisons. For expected frequencies between 1 and 5 with a total sample size of 40 or more, Yates’ correction for continuity was applied. Kaplan–Meier survival analysis was used to describe the survival curves, and the log-rank test was applied to assess the differences between them. Statistical significance was set at P < 0.05.

## 3 Results

### 3.1 Comparison of clinicopathological characteristics between the two groups

There were no statistically significant differences in age, body mass index (BMI), PSA levels, high blood pressure (HBP), diabetes mellitus (DM), smoking history, Gleason score, pre-enrollment abiraterone duration, pre-enrollment enzalutamide duration, or extent of disease (EOD) score between the DP+MDV 3100 and DP groups before treatment (P > 0.05), as shown in **Table 1**.

**Table 1:** Comparison of pre-treatment clinicopathological characteristics between the two groups.

Characteristics	DP+MDV3100	DP	P value
n	38	29	
Age (yr), mean ± sd	73.079 ± 7.7543	72.31 ± 7.0766	0.678
BMI (kg/m^2^), mean ± sd	23.334 ± 2.8077	24.038 ± 2.7547	0.310
HBP, n (%)			0.180
Yes	22 (57.9%)	12 (41.4%)	
No	16 (42.1%)	17 (58.6%)	
DM, n (%)			0.310
Yes	10 (26.3%)	11 (37.9%)	
No	28 (73.7%)	18 (62.1%)	
Smoking, n (%)			0.353
Yes	22 (57.9%)	20 (69%)	
No	16 (42.1%)	9 (31%)	
Initial PSA (ng/mL), median (IQR)	54.05 (37.288, 92.377)	50.23 (24.81, 109.26)	0.400
Gleason Score, n (%)			0.836
3 + 3	1 (2.6%)	1 (3.4%)	
3 + 4	8 (21.1%)	4 (13.8%)	
3 + 5	0 (0%)	1 (3.4%)	
4 + 3	5 (13.2%)	3 (10.3%)	
4 + 4	8 (21.1%)	9 (31%)	
4 + 5	12 (31.6%)	8 (27.6%)	
5 + 3	1 (2.6%)	0 (0%)	
5 + 4	1 (2.6%)	2 (6.9%)	
5 + 5	2 (5.3%)	1 (3.4%)	
Pre-enrollment abiraterone duration(month), median (IQR)	26 (20.25, 31.75)	25 (17, 33)	0.924
Pre-enrollment enzalutamide duration(month), median (IQR)	6 (4, 8)	6 (5, 8)	0.893
Pre-enrollment EOD score, n (%)			0.399
1	20 (55.56%)	14 (70.00%)	
2	14 (38.89%)	6 (30.00%)	
3	2 (5.56%)	0 (0.00%)	

BMI, body mass index; HBP, high blood pressure; DM, diabetes mellitus; PSA, prostate-specific antigen; IQR, interquartile range; DP, docetaxel + prednisone; MDV3100, enzalutamide; EOD, extent of disease.

### 3.2 Comparison of treatment effectiveness between the two groups

In DP+MDV3100 group, 36 patients experienced a PSA decline exceeding 50% from baseline, compared with 21 patients in the DP group. The proportion of patients with a PSA decline >50% was higher in the study group than that in the control group (P < 0.05). Additionally, improvements in bone metastases were observed in 36 patients and lymph node metastases in 35 patients in the DP+MDV3100 group, which were significantly higher than those in the DP group (P < 0.05), as shown in **Table 2**.

**Table 2:** Comparison of treatment outcomes between the two groups.

Characteristics	DP+MDV3100	DP	P value
n	38	29	
PSA decline ≥ 50%, n (%)			0.028
Yes	36 (94.7%)	21 (72.4%)	
No	2 (5.3%)	8 (27.6%)	
Bone metastasis regression, n (%)			0.013
Yes	36 (94.7%)	20 (69%)	
No	2 (5.3%)	9 (31%)	
Lymph node metastasis regression, n (%)			0.014
Yes	35 (92.1%)	20 (69%)	
No	3 (7.9%)	9 (31%)	

PSA, prostate-specific antigen; DP, docetaxel + prednisone; MDV3100, enzalutamide.

### 3.3 Comparison of changes in VAS scores before and after treatment between the two groups

Before treatment, there was no statistically significant difference in VAS scores between the two groups (P > 0.05). After treatment, both groups showed significant improvements in VAS scores (P < 0.05). However, the post-treatment VAS scores were lower in the intervention group than in the control group (P < 0.05) (**Table 3**).

**Table 3:** Comparison of VAS score changes before and after treatment between the two groups.

Characteristics	DP+MDV3100	DP	P value
n	38	29	
Pre-treatment VAS, median (IQR)	6 (4, 7)	6 (4, 7)	0.832
Post-treatment VAS, median (IQR)	2 (1, 3)	3 (3, 5)	<0.001

VAS, visual analog scale; IQR, interquartile range; DP, docetaxel + prednisone; MDV3100, enzalutamide.

### 3.4 Comparison of PFS between the two groups

**Figure 1:**
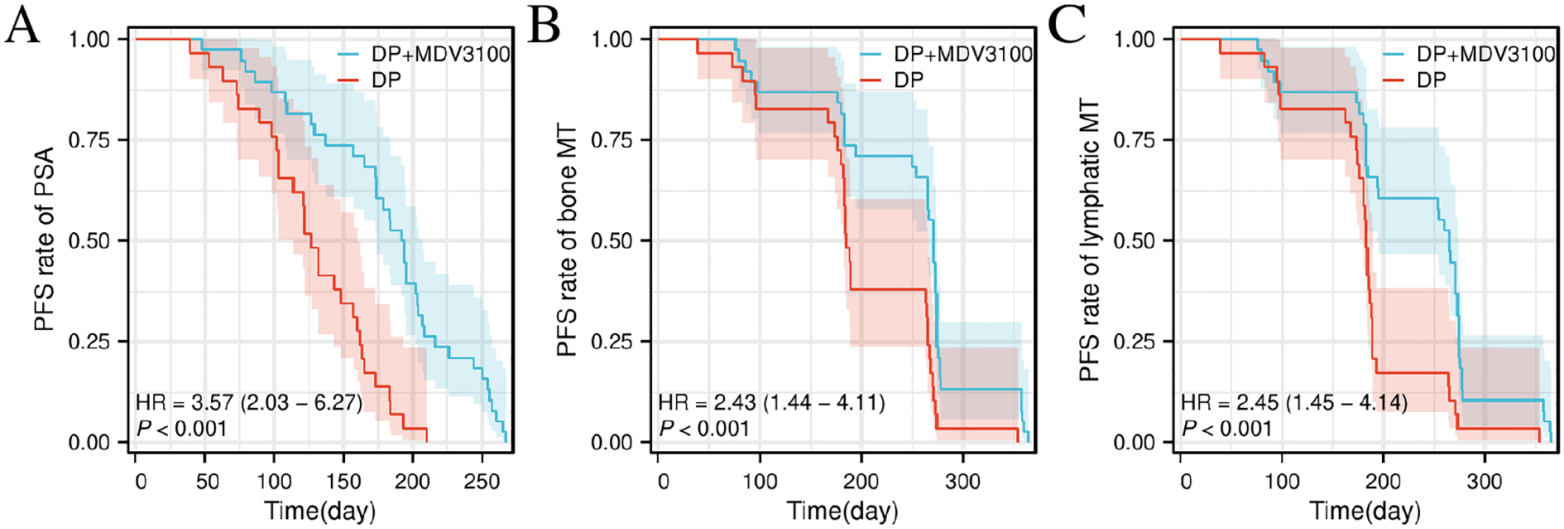
Comparison of disease PFS Kaplan–Meier curves between the two groups. **(A)** Comparison of PFS between Group DP+MDV3100 and Group DP. **(B)** Comparison of bone metastasis-specific PFS between Group DP+MDV3100 and Group DP. **(C)** Comparison of lymph node metastasis-specific PFS between Group DP+MDV3100 and Group DP. PSA, prostate-specific antigen; HR, Hazard Ratio; MT, metastasis; PFS, progression-free survival; DP, docetaxel + prednisone; MDV3100, enzalutamide.

Among the patients with improved treatment outcomes, the median PSA PFS in the Group DP+MDV3100 was 193 days (95% confidence interval [CI]: 174–207 days), which was longer than the 127 days (95% CI: 114–160 days) observed in the DP group. The median PFS for bone metastases was 271 days (95% CI: 265–274 days), which exceeded the 185 days in the control group (95% CI: 183–265 days). Similarly, the median PFS for lymph node metastases was 265 days (95% CI: 194–274 days) compared to 183 days (95% CI: 180–189 days) in the control group (P < 0.05), as illustrated in **Figure 1**.

### 3.5 Comparison of adverse reactions between the two groups

In the Group DP+MDV3100, all 38 patients experienced adverse reactions. The most common symptoms were myelosuppression, followed by malaise, gastrointestinal responses, lumbago, backache, abnormal liver function, edema, neurological symptoms (characterized by numbness in the extremities), rash, and high blood pressure. No Grade III or higher adverse reactions were observed, and no patient discontinued treatment due to adverse effects. In the DP group, all 29 patients experienced adverse reactions, with myelosuppression being the most common, followed by gastrointestinal responses, malaise, abnormal liver function, neurological symptoms (characterized by numbness in the extremities), and edema. No Grade III or higher adverse reactions were observed, and no patient discontinued treatment due to adverse effects. Notably, the study group had a higher incidence of malaise, lumbago, and backache than the control group (P < 0.05), as shown in **Table 4**.

**Table 4:** Comparison of adverse reactions between the two groups.

Characteristics	DP+MDV3100	DP	P value
n	38	29	
myelosuppression, n (%)			0.224
1	25 (65.8%)	23 (79.3%)	
2	13 (34.2%)	6 (20.7%)	
malaise, n (%)			< 0.001
0	7 (18.4%)	22 (75.9%)	
1	25 (65.8%)	6 (20.7%)	
2	6 (15.8%)	1 (3.4%)	
gastrointestinal responses, n (%)			0.938
0	22 (57.9%)	17 (58.6%)	
1	14 (36.8%)	11 (37.9%)	
2	2 (5.3%)	1 (3.4%)	
abnormal liver function, n (%)			0.966
0	31 (81.6%)	23 (79.3%)	
1	6 (15.8%)	5 (17.2%)	
2	1 (2.6%)	1 (3.4%)	
edema, n (%)			0.464
0	32 (84.2%)	27 (93.1%)	
1	6 (15.8%)	2 (6.9%)	
neurological symptoms, n (%)			0.775
0	32 (84.2%)	26 (89.7%)	
1	6 (15.8%)	3 (10.3%)	
lumbago and backache, n (%)			0.031
0	30 (78.9%)	29 (100%)	
1	7 (18.4%)	0 (0%)	
2	1 (2.6%)	0 (0%)	
rash, n (%)			0.596
0	36 (94.7%)	29 (100%)	
1	2 (5.3%)	0 (0%)	
HBP, n (%)			0.596
0	36 (94.7%)	29 (100%)	
1	2 (5.3%)	0 (0%)	

HBP, high blood pressure; DP, docetaxel + prednisone; MDV3100, enzalutamide.

### 3.6 Ethics statement

The investigators affirmed comprehensive accountability for the methodological rigor and validity of this research. Rigorous validation processes were implemented to address potential quality concerns throughout the study lifecycle. This study was approved by the Institutional Review Board of the First Affiliated Hospital of Soochow University (Protocol ID: 511/2024) and strictly adhered to the ethical principles established in the 2013 revision of the Declaration of Helsinki. Written informed consent was obtained from all enrolled subjects prior to trial initiation.

## 4 Discussion

The mCRPC stage marks an advanced phase of PCa and significantly reduces patient survival (10). Historically, docetaxel has been regarded as the only drug capable of extending survival in patients with mCRPC (15). It primarily exerts antitumor effects by affecting microtubules, thereby disrupting mitosis and inducing apoptosis (16). However, docetaxel may also benefit patients with prostate cancer through androgen receptor signaling pathways and the inhibition of nuclear translocation (17). Abiraterone is a selective and irreversible CYP17 inhibitor that blocks androgen synthesis from all sources (18). Furthermore, enzalutamide acts by inhibiting the binding, nuclear translocation, and activity of the androgen receptor (19). Both abiraterone and enzalutamide are NHTs that target androgen activity and may lead to cross-resistance, resulting in suboptimal outcomes when switching from abiraterone to enzalutamide (12). However, research indicates that using enzalutamide after abiraterone resistance improves tumor PFS but not overall survival (20). While the mechanism of action of docetaxel shares some similarities with that of enzalutamide, two major studies have demonstrated significant benefits in patients with mCRPC when enzalutamide is used before or after docetaxel (21, 22). Based on these findings and patient preferences, the practice of administering docetaxel chemotherapy following sequential treatment with abiraterone and enzalutamide in patients with mCRPC has become more prevalent than immediately switching to chemotherapy after a single NHT treatment.

The above information suggests that while different drugs have overlapping mechanisms of action, combination therapy may provide better outcomes compared to monotherapy. The PRESIDE study was a two-phase, multinational, double-blind, randomized, placebo-controlled, phase 3b trial conducted at 123 European sites (23). In the first phase, 688 patients with mCRPC received 160 mg enzalutamide daily alongside ADT. After 13 weeks, 271 patients who showed a PSA decline and subsequent progression (radiographic or PSA progression) entered the second phase. All patients underwent 10 cycles of docetaxel chemotherapy (75 mg/m² every 3 weeks) and daily prednisone (10 mg) and were randomly assigned (1:1) to receive either 160 mg/day enzalutamide or a placebo. Results showed that the enzalutamide group had a median tumor PFS of 9.5 months (95% CI: 8.3–10.9 months) compared to the 8.3 months (95% CI: 6.3–8.7 months) in the placebo group (hazard ratio = 0.72, 95% CI: 0.53–0.96, P = 0.027). This indicates that enzalutamide combined with docetaxel could extend tumor PFS in enzalutamide-responsive mCRPC patients beyond that achieved by docetaxel alone. The CHEIRON study involved untreated patients with mCRPC who were randomly assigned (1:1) to receive eight cycles of docetaxel chemotherapy with or without enzalutamide (similar to the PRESIDE protocol) (24). Patients in the combination group exhibited better progression rates after 6 months compared to the docetaxel-only group. These studies highlight the significance of the enzalutamide-docetaxel combination in mCRPC treatment. Unlike the CHEIRON and PRESIDE studies, which did not include patients previously treated with abiraterone, the present study focused on patients with mCRPC who progressed after abiraterone followed by enzalutamide, aligning better with current clinical practices. The study found that even after dual NHT treatment, continuing enzalutamide with docetaxel was more effective than docetaxel alone. The analysis demonstrated that more patients in the combination therapy group experienced >50% PSA decline and improvements in bone and lymph node metastases, highlighting the advantages of combination therapy. Continued follow-up indicated that most patients experienced PSA or imaging progression within one year of treatment. Survival analysis demonstrated more significant benefits for PSA levels and metastasis control with combination therapy, suggesting a synergistic effect between enzalutamide and docetaxel.

Pain is a significant factor that affects the quality of life of patients with advanced malignancies. Both the study and control groups showed a reduction in the VAS scores after treatment, with the study group achieving lower VAS scores, indicating better pain control with combination therapy. This improvement is in line with the greater resolution of bone metastasis and extended PFS in the study group. Clinically, effective medication and care strategies are essential to alleviate pain and enhance patients’ quality of life (25). In terms of adverse reactions, both groups experienced significant myelosuppression, though no Grade III or higher toxicities was observed. Unlike enzalutamide studies, in which myelosuppression was less pronounced, Malinowski et al. (26) indicated that nearly all docetaxel regimens for mCRPC resulted in hematological toxicity, including Grade III-IV neutropenia (12%–15%) and febrile neutropenia (6%–12%). Widespread myelosuppression observed in both groups was likely attributable to docetaxel. Other adverse reactions in the study group included malaise, gastrointestinal responses, lumbago, and backache, consistent with the known side effects of enzalutamide (27). Additional mild adverse effects included liver dysfunction, neurological symptoms, edema, rash, and HBP; however, none necessitated treatment discontinuation. In the control group, aside from myelosuppression, the main adverse reactions were gastrointestinal responses and malaise, with other effects such as liver dysfunction, edema, and neurological symptoms remaining mild (Grades I-II). Overall, although combining enzalutamide with docetaxel introduces enzalutamide-specific adverse effects, these are generally mild and do not require discontinuation or alteration of the treatment regimen, offering an advantage over the use of docetaxel alone.

This study has several limitations that should be acknowledged. First, the follow-up duration was relatively short, which restricted our ability to fully evaluate long-term outcomes such as overall survival (OS). We plan to continue follow-up in future research to address this limitation. Second, as a retrospective study, treatment allocation was determined by physician and patient preference rather than randomization, which may have introduced selection bias and increased the risk of confounding factors affecting treatment outcomes. Third, molecular profiling and biomarker analyses were not performed in this study; however, we intend to conduct further research to evaluate the sensitivity of patients with different genotypes to various treatments. Finally, although pain was assessed using the VAS, other standardized quality of life assessments were not included. In future studies, we will incorporate more comprehensive quality of life measures to provide a deeper understanding of patient-reported outcomes. These limitations underscore the need for prospective, randomized studies with longer follow-up, molecular analyses, and more comprehensive quality of life assessments in future research.

In summary, this study demonstrated that the combination of enzalutamide and docetaxel offers advantages over docetaxel alone in controlling PSA levels and bone metastases in patients with mCRPC who progressed after effective treatment with abiraterone followed by enzalutamide. This combination also helps to alleviate pain symptoms and is associated with relatively mild adverse effects. However, this study was limited by its small sample size and short follow-up period, indicating the need for further analysis and validation with a larger patient cohort.

## Data Availability

The datasets presented in this study can be found in online repositories. The names of the repository/repositories and accession number(s) can be found below: https://figshare.com/s/2a15a646e7dc713e9830.
